# Frequent cough in unsatisfactory controlled asthma – results from the population-based West Sweden Asthma Study

**DOI:** 10.1186/1465-9921-15-79

**Published:** 2014-08-18

**Authors:** Roxana Mincheva, Linda Ekerljung, Anders Bjerg, Malin Axelsson, Todor A Popov, Bo Lundbäck, Jan Lötvall

**Affiliations:** Krefting Research Centre, University of Gothenburg, SE 40530 Göteborg, Sweden; Faculty of Health and Society, Malmö University, SE-205 06 Malmö, Sweden; Clinic of Allergy and Asthma, Medical University of Sofia, BG 1431 Sofia, Bulgaria

**Keywords:** Cough prevalence, Level of asthma control, Lung function, Population-based study

## Abstract

**Background:**

Asthma is a complex disease presenting with variable symptoms which are sometimes hard to control. The purpose of the study was to describe the prevalence of asthma symptoms, use of asthma medications and allergic sensitization in subjects with asthma. We also related those indices to the level of asthma control, lung function and in particular, cough.

**Methods:**

An extensive questionnaire was sent to randomly selected adults from the West Sweden region. Clinical examinations and interview were performed in a subset. Of the participants, 744 were defined as having an ongoing asthma - reported ever having asthma or physician diagnosed asthma and one of the following – use of asthma medications, recurrent wheeze or attacks of shortness of breath with or without wheeze in the last 12 months. A respiratory disease-free control group of 847 subjects was also described.

**Results:**

According to GINA guidelines, 40.6% of the asthmatics had partly controlled and 17.8% had uncontrolled asthma. Asthmatic subjects reported significantly more symptoms in the last 12 months than the control group – wheezing (79.4 *vs* 9.2%), shortness of breath (36.1 *vs* 2.5%), wheezing with shortness of breath (58.7 *vs* 1.3%). Important complaints were morning cough (42.5 *vs* 15.5%), cough with sputum production (36.1 vs 6.8%) and longstanding cough (32.5 *vs* 11.1%), which bothered two thirds of the uncontrolled and one third of partly controlled subjects. Asthma medications were used by 87.5% of the asthmatics, although around 30% of them who had insufficiently controlled disease used only short-acting beta-agonists. Asthmatics also had lower lung function, reacted to lower doses of methacholine that the controls and 13.6% of them had a FEV1/FVC ratio below 0.7. Allergic rhinitis was reported by 73.8% of the asthmatics and they were more frequently sensitized to several common allergens.

**Conclusions:**

Approximately 60% of asthmatics from this population-based study had insufficiently controlled asthma and persistent complaints, despite a high use of asthma medications. These self-reported symptoms were supported by clinical examination data. Increased cough frequency is an indicator of a more severe and difficult to control disease and should be considered when asthma is characterized.

## Background

Asthma is a complex syndrome with variable clinical course and presentation which is estimated to trouble more than 300 million people worldwide [1]. An increase in asthma prevalence and morbidity was noted from the middle of the last century [2–4] but this tendency seems to have subsided in the last decade, at least in the Westernised countries. [5–7]. A recent large-scale epidemiological survey in Sweden revealed an estimated prevalence of asthma around 8.5% suggesting a steady level for the last two decades [5].

Asthma is classically characterized by variable episodes of shortness of breath, chest tightness, wheezing and cough which can concur or be present at different times. Complementary lung function tests often show decreased forced expiratory volume in 1 second (FEV1), increased airway responsiveness to bronchoconstrictive agents like methacholine and reversibility of more than 12% and 200 ml after inhalation of a bronchodilator during periods with asthma symptoms [8]. Even though breathlessness and wheeze are more frequent asthma symptoms, cough often seems to be the most troublesome and problematic complaint for the patient. It also tends to be more resilient to treatment with bronchodilators than the other complaints [9]. Cough often presents as a longstanding symptom, even if the disease is adequately controlled by anti-inflammatory treatment, and recently it was shown that transient receptor potential vanilloid 1 channels are overexpressed in the bronchial epithelium of more severe asthma [10, 11]. Moreover, cough might be associated with airway hyperreactivity without the presence of dyspnea, wheezing and airflow obstruction, an entity that has been coined as cough-variant asthma [12]. Asthma is often accompanied by several comorbidities like allergic rhinitis (AR) and chronic rhinosinusitis (CRS) which are also considered to be substantial risk factors for asthma development [13–16] and the latter a predictor for more severe disease [17, 18]. AR and CRS also cause dripping of secretions back to the nasopharynx leading to perpetuation of cough symptoms [19, 20]. Today’s mainstream asthma treatment is guided by the early use of anti-inflammatory medications and the increased need for reliever medications is considered an indicator of inadequately controlled disease [21]. Guidelines suggest the use of short-acting beta-2 agonists (SABA) alone should be given exclusively to a small group of asthmatics with intermittent disease.

The aim of the study was to determine the prevalence of respiratory symptoms, the use of asthma medications, the presence of concomitant diseases and the level of asthma control in the subset of subjects from the West Sweden Asthma Study with asthma, and to relate these to lung physiology and cough, with the hypothesis that these are interconnected. The understanding of such associations is important to dissect mechanisms of asthma severity.

## Methods

### Study population, questionnaire and clinical examinations

**Figure 1:**
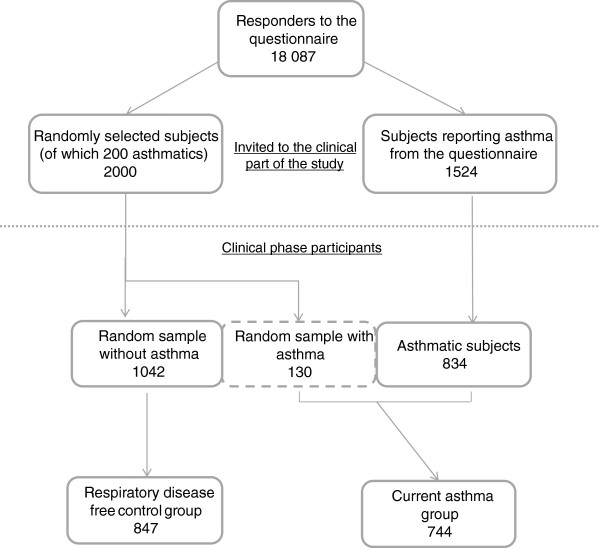
**Sampling procedure of defining the asthma and respiratory disease-free control groups.**

The grounds for the sample selection were based on a large scale population study, initiated in 2008 in West Sweden that is described in details elsewhere [5]. The sampling procedure is depicted on Figure 1. In short, an extensive postal questionnaire was sent to 30 000 people, randomly selected from the West Sweden region, aged 16-75 years, of which 18 087 participated. It comprised of the OLIN- questionnaire and a Swedish version of the GA^2^LEN questionnaire and additionally of questions about environmental and occupational exposures as well as family background and socioeconomic status. From the responders, a randomly selected sample of 2000 subjects as well as all those who reported ever asthma with current symptoms or asthma medication or physician diagnosed asthma (n = 1524) were invited to take part in the subsequent clinical phase of the study. Altogether, 2006 subjects agreed to participate in the clinical part which was conducted between 2009 and 2012.

The clinical part consisted of a detailed interview about asthma presence, asthma symptoms and medications, exacerbations, health-care and emergency visits, concurrent obstructive and non-obstructive pulmonary as well as extra-pulmonary diseases, smoking and asthma triggers. Clinical examinations included physical check-up, spirometry before and after a bronchodilator, fractional exhaled nitrogen oxide (FeNO) measurement, methacholine testing for bronchial hyperresponsiveness, skin-prick tests to 11 common allergens (*D. Pteronyssinus, D. farina,* Alternaria, Cladosporium, German cockroach, dog, cat, horse, timothy, mugwort, birch) and blood testing. The focus of the present study was a subset of these subjects (n = 744) who were defined as having ongoing asthma - reported ever having asthma or physician diagnosed asthma and one of the following in the last 12 months – 1) use of asthma medications, 2) recurrent wheeze or 3) attacks of shortness of breath with or without wheeze. This is a slightly more severe group than sometimes described in epidemiology as “current asthma”, which would include also individuals with any wheeze in the last year. The reported symptoms and objective measurements were compared with a group of respiratory disease – free (RDF) subjects (n = 847) – those not having reported ever or physician diagnosed asthma or asthma symptoms and use of asthma medications, COPD, chronic bronchitis, emphysema or tuberculosis and chronic productive cough.

The study was approved by the local Ethics Committee of University of Gothenburg.

### Definitions

Definitions are based on the questions from the interview:

*Ever asthma*:” Have you ever had asthma?”; *Physician-diagnosed asthma*:” Have you been diagnosed as having asthma by a doctor?”; *Asthma medications*:” Have you used asthma medications (permanently or as needed) in the last 12 months?”; *Shortness of breath*;”Have you ever had suddenly appearing shortness of breath or breathlessness?” *and* “have you had these symptoms in the last 12 months?”; *Recurrent wheeze*:” Do you usually have wheezing or whistling in the chest when breathing?”; *Any wheeze*:” Have you had whistling or wheezing in the chest on any occasion during the last 12 months?”; *Wheezing with shortness of breath*: Yes to *Any wheeze* and”Have you been at all breathless when the wheezing or whistling was present?”; *Longstanding cough*:” Have you had a longstanding cough during the last 12 months?”; *Morning cough*: “Do you usually cough or hawk in the mornings?”, *Sputum production*:” Do you usually cough or hawk phlegm from the chest, or do you feel like you have phlegm in the chest that is difficult to bring up by coughing and hawking?”; *Sputum production longer than 3 months per year*:” Do you cough of hawk phlegm (or do you have phlegm which is difficult to bring up despite coughing) most days in periods of at least 3 months per year?”; *Allergic rhinitis*:” Do you currently have or have you ever had allergic nose problems of hay fever?”; *Physician – diagnosed COPD, chronic bronchitis, emphysema, tuberculosis*:” Have you been diagnosed as having COPD, chronic bronchitis, emphysema or tuberculosis by a physician” (asked in separate questions); *Doctor’s office visits*:” Have you consulted a physician or other medical care because of breathlessness, shortness of breath or whistling in the chest, cough with or without phlegm, phlegm in the chest or other lung or airway problems in the last 12 months?”; *Hospitalisations*: “Have you been hospitalised due to problems with your breathing during the last 12 months?”; *Emergency unit visits*:” Have you visited the emergency unit because of problems with your breathing during the last 12 months?”; *Level of asthma control* – *controlled, partly controlled and uncontrolled (according to GINA 2006 guidelines*) based on the presence of daytime/nighttime symptoms, limitation of daily activities, use of reliever medications, pulmonary function and exacerbations, evaluated by the person conducting the interview. With regard to GINA guidelines patients were categorised as having controlled asthma when they had no limitation of daily activities, nocturnal symptoms and exacerbations, had none or twice or less a week of daytime symptoms and need for rescue medications as well as normal lung function (FEV1 > 80% predicted). Partly controlled were patients having any of the following – limitation of activities, nocturnal symptoms/awakenings, more than twice a week had symptoms during the day and required reliever medication, had one or more exacerbation per year and FEV1 below 80% predicted. All subjects who reported three or more of the features of partly controlled disease or exacerbation in any week were regarded as uncontrolled asthmatics.

### Analyses

Statistical analyses were performed using IBM SPSS Statistics for Windows, Version 21.0. Armonk, NY: IBM Corp. Descriptive statistics (frequencies, means and standard deviation) were acquired to describe the characteristics of the subjects. Proportions were compared with chi-square test and Mantel-Haenszel test for trend was used whenever appropriate. Continuous variables were analysed using unpaired *t*-test. P-value of <0.05 was regarded as statistically significant. Adjusted logistic regression analyses were performed to determine risk factors, presented as odds ratios (OR) with 95% confidence intervals (95% CI).

## Results

### General characteristics

The general characteristics of the participants in the study as well as smoking status and body mass index are presented in Table 1. The studied populations did not differ in terms of smoking status. As for BMI there were significantly more asthmatics in the range of obesity i.e. having BMI above 30 (25.4% *vs* 12.8%, p < 0.001) than controls. Also, the women tend to be more represented in the asthmatic sample (61.8% *vs* 53.2% in the RDF controls sample) but this is in accordance with the literature data about sex difference in asthma prevalence [22]. Furthermore, risk factor analysis showed that female gender and BMI > 30 increase the risk of having uncontrolled asthma (Table 2).

**Table 1:** **Age, sex, BMI and smoking status of the asthmatics and the controls**

Variables	No	Current asthma No (%)	No	Controls No (%)	P values
Number of subjects	744		847		
Sex (women)		460 (61.8)		451 (53.2)	0.001
Age		47.6 ± 15.5		49.9 ± 15.5	0.002
BMI	743		847		
<20		23 (3.1)		38 (4.5)	0.150
20-25		242 (32.6)		337 (39.8)	0.003
25-30		289 (38.9)		364 (43.0)	0.099
≥30		189 (25.4)		108 (12.8)	<0.001
Smoking status	742		844		
Non-smokers		386 (52)		457 (54.1)	0.206*
Ex-smokers		261 (35.2)		298 (35.3)	
Smokers		95 (12.8)		89 (10.5)	

Data are presented as No (%), except for age presented as mean ± SD. Chi-square tests are used to make comparisons between individual variables. *p-value from the test for trend.

**Table 2:** **Adjusted logistic regression showing risk factors (Odds Ratios (95**% **confidence intervals)) associated with party controlled and uncontrolled asthma**

		Partly controlled asthma	Uncontrolled asthma
		OR (95% CI)	OR (95% CI)
Age	Increasing	**1.022 (1.006 – 1.038)**	**1.037 (1.013 – 1.062)**
Gender	Female	**1.663 (1.118 – 2.475)**	**2.231 (1.240 – 4.012)**
Atopy	Yes	0.799 (0.512 – 1.248)	0.868 (0.467 – 1.613)
BMI	Below 20	1.265 (0.444 – 3.604)	1.040 (0.266 – 4.071)
	25-30	1.059 (0.675 – 1.660)	1.087 (0.636 – 1.856)
	Above 30	1.206 (0.708 – 2.054)	**2.543 (1.455 – 4.447)**
Longstanding cough	Yes	**2.263 (1.438 – 3.562)**	**4.182 (2.332 – 7.497)**
Productive cough	Yes	**2.991 (1.888 – 4.738)**	**6.691 (3.575 – 12.524)**
Morning cough	Yes	**2.075 (1.379 – 3.121)**	**4.679 (2.635 – 8.311)**

Significant risk factors are highlighted in bold. Reference categories were: male gender, BMI 20-25, having no longstanding, productive and morning cough, respectively.

### Respiratory symptoms

**Figure 2:**
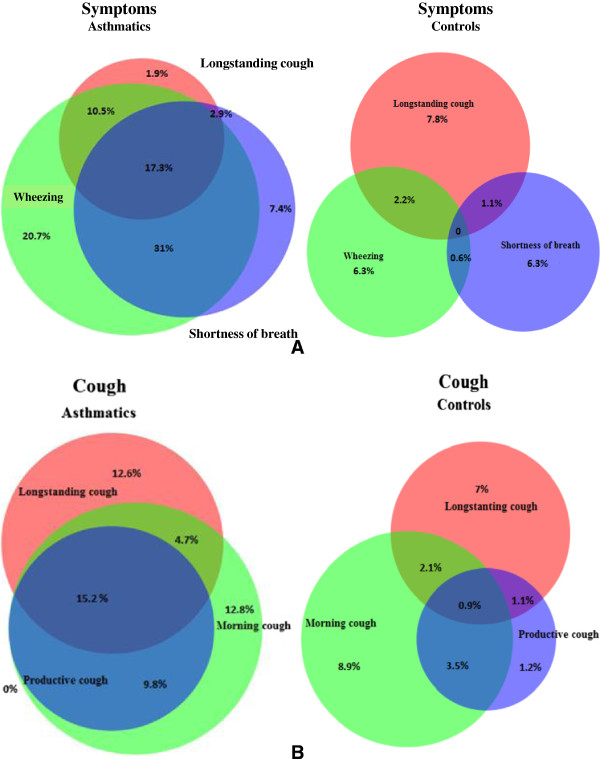
**A Prevalence of cough, shortness of breath and wheezing in asthma (n = 744) and respiratory disease-free control group (n = 847), B Prevalence of cough complaints in asthmatics (n = 744) and respiratory disease - free control group (n = 847).**

The respiratory symptoms reported in the last 12 months were significantly pronounced in the asthmatic group compared to the RDF group (Table 3). The most prevalent symptoms were wheezing (79.2% *vs* 9.2%), wheezing with shortness of breath (58.7% *vs* 1.3%) and morning cough (42.5% *vs* 15.5%), followed by productive cough (36.1% *vs* 6.8), shortness of breath (36.1% *vs* 2.5%) and longstanding cough (32.5% *vs* 11.1%), all significant at p < 0.001. Additionally, when longstanding or morning cough were evaluated in the absence of shortness of breath and wheezing, asthmatic subjects were significantly less than the RDF control group (1.9% *vs* 7.8% and 3.1% *vs* 11.2%, respectively) (Figure 2a). This observation accounts for the multilayered nature of the asthmatic condition and poses question on the appraisal of a disease presenting only with cough. When reporting different types of cough, asthmatics have clustered complaints (Figure 2b), and report having productive cough always coupled with longstanding and/or morning cough. We also found that productive, morning and longstanding cough increased the risk of having partly or uncontrolled disease (Table 2). Control subjects, on the other hand, have less frequent complaints and tend to report them as separate symptoms, with very few (0.9%) having morning cough, productive cough and longstanding cough at the same time.

**Table 3:** **Prevalence of respiratory symptoms reported in the last 12 months in the groups of the asthmatics and controls**

Variables	No	Current asthma No (%)	No	Controls No (%)	P values
Symptoms	744		847		
Longstanding cough		242 (32.5)		94 (11.1)	<0.001
Morning cough		316 (42.5)		131 (15.5)	<0.001
Sputum production		268 (36.1)		57 (6.8)	<0.001
Wheezing		591 (79.4)		78 (9.2)	<0.001
Wheezing with shortness of breath		437 (58.7)		11 (1.3)	<0.001
Shortness of breath		269 (36.1)		21 (2.5)	<0.001
Longstanding cough without other symptoms		14 (1.9)		66 (7.8)	<0.001
Morning cough without other symptoms		23 (3.1)		95 (11.2)	<0.001
Allergic rhinitis and longstanding cough		170 (22.8)		34 (4.0)	<0.001
Allergic rhinitis		548 (73.8)		259 (30.7)	<0.001

Data are presented as No (%). Chi-square tests are used to make comparisons between individual variables.

Allergic rhinitis is reported in 73.8% of the asthmatic subjects, compared to 30.7% of the RDF control group, and allergic rhinitis and cough are present in 22.8% of the asthmatics but in only 4% of the controls.

### Lung function and sensitisation

**Figure 3:**
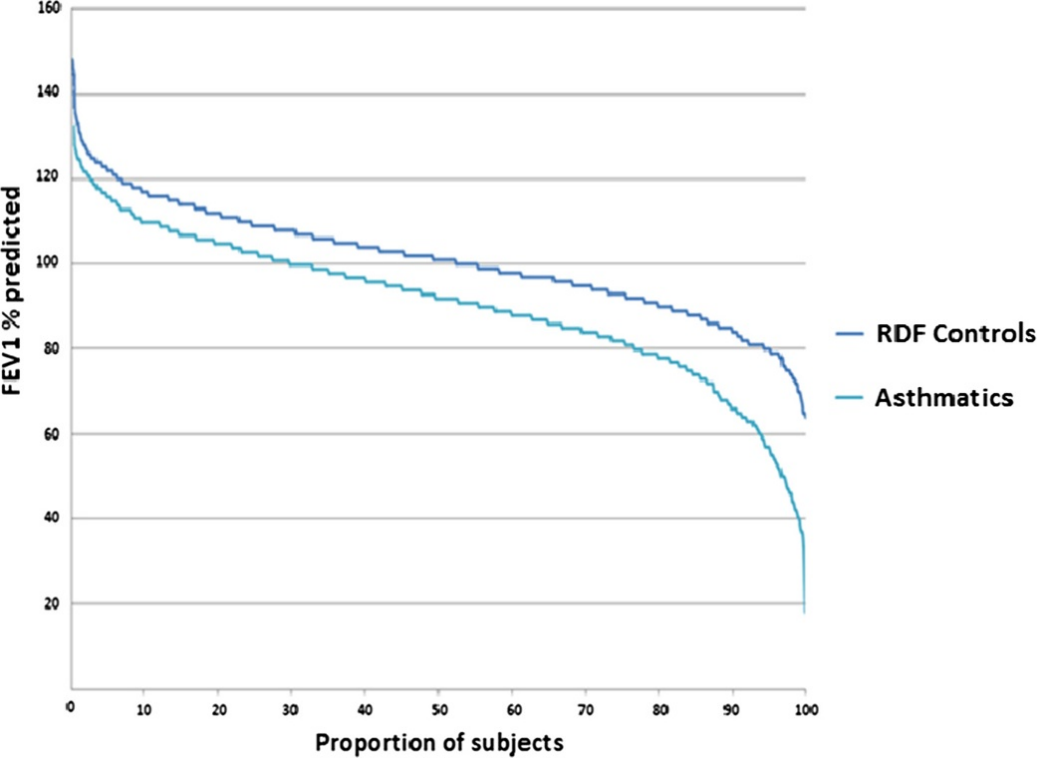
**Difference in FEV1% predicted between current asthmatics and RDF controls.** Asthmatics have distinctly lower lung parameters than the control group.

**Figure 4:**
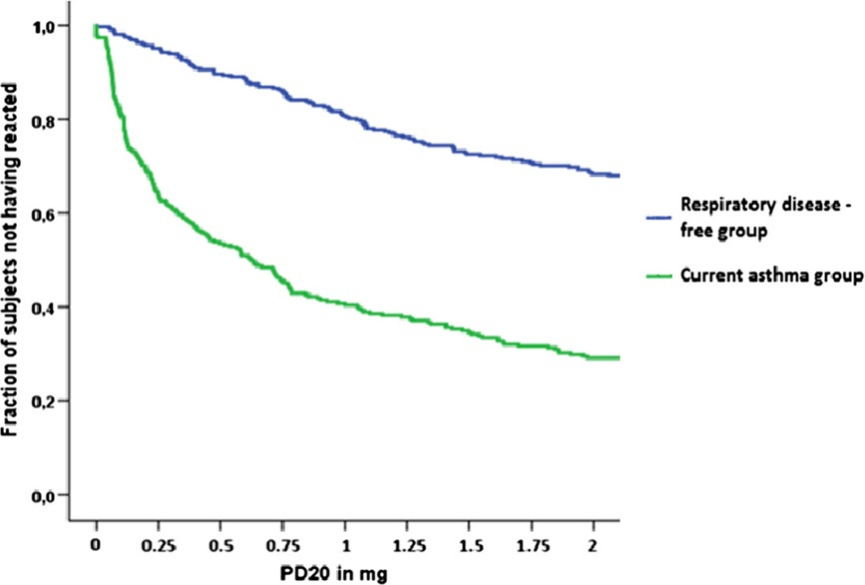
**Fraction of current asthmatics and respiratory disease-free controls who have not reacted to cut-off values for positive methacholine reactivity.**

The results from the pulmonary function tests, skin-prick tests and health-care visits are summarised in Table 4. The mean value of pre-bronchodilator FEV1% predicted in the asthmatics is 90.6 ± 17.6 (standard deviation) versus 101 ± 12.9 in the control group (Figure 3). Increase of the FEV1 with ≥ 12% and 200 ml after inhalation of a bronchodilator was noted in 24.8% of the asthmatics and 3.4% in the RDF controls. Low lung function, estimated as having FEV1 below 80% predicted, was present in 23.2% in the asthma group, but only is 5.3% in the respiratory disease-free group. Proportion of asthmatics that react with decrease of the FEV1 more than 20% after inhaling a cumulative dose of 1.96 mg of methacholine is 70.9%, but still 32% of the control group also shows increased reactiveness of their airways (Figure 4).

**Figure 5:**
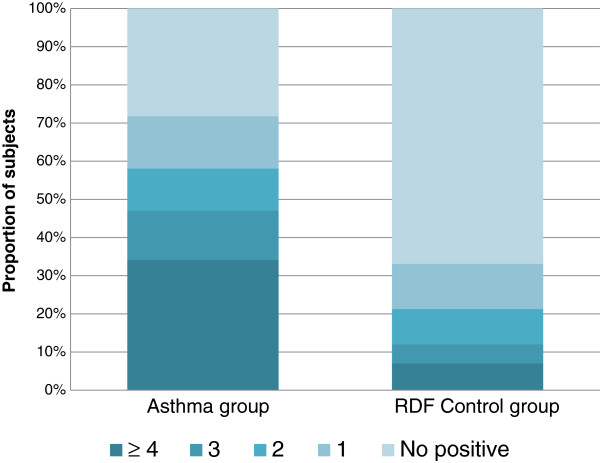
**Prevalence of the sensitisation to a number of common allergens in current asthmatics (n = 575) and RDF controls (n = 577).** Asthmatics have more pronounced sensitisation to polyallergens.

Subjects with asthma that had at least one positive skin-prick test were 71.8% of those who had the test performed (575 subjects) and 33.1% of the controls showed positive results (from 577 subjects tested). Moreover, when sensitisation was present in the asthmatics it was often to four or more allergens highlighting a noticeable polysensitisation in comparison to the respiratory disease-free subjects (Figure 5).

**Table 4:** **Description of lung function, sensitisation and health-care utilisation**

Variables	No	Current asthma	No	Controls	P values
Overall number of subjects	744		847		
FEV1% predicted	707	90.6 ± 17.6	816	101.0 ± 12.9	<0.001
FEV1% predicted below 80%	707	164 (23.2)	816	43 (5.3)	<0.001
Positive reversibility after a bronchodilator	444	110 (24.8)	465	16 (3.4)	<0.001
FEV1/FVC ratio < 0.7	705	96 (13.6)	817	33 (4.0)	<0.001
FeNO ppb^#^	597	26.88 ± 24.3	676	19.03 ± 11.0	<0.001
Reactiveness to PD20 ≤ 1.96 mg**	275	195 (70.9)	328	105 (32.0)	<0.001
At least 1 positive SPT	575	413 (71.8)	577	191 (33.1)	<0.001
Number of positive SPT in the sensitised subjects	413		191		
1		79 (19.1)		68 (35.6)	<0.001*
2		63 (15.3)		54 (28.3)	
3		75 (18.2)		29 (15.2)	
≥4		196 (47.4)		40 (20.9)	
Doctor's office visits	740	263 (35.3)	844	80 (9.5)	<0.001
Hospitalisations	735	16 (2.2)	844	1 (0.1)	<0.001
Emergency visits due to respiratory problems	733	78 (10.6)	842	11 (1.3)	<0.001

Comparisons of FEV1/FVC ratio, PD20 reactiveness, positive reversibility after a bronchodilator and BMI are done using Chi-square test, other clinical parameters are compared using unpaired *t*-test. *p-value from the test for trend.

^**#**^measured at 50 ml/s.

^**^% of subjects reacted to methacholine dose ≤ of 1.96 mg with 20% fall in the FEV1.

Data are presented as number of subjects No (%) or mean ± SD.

### Health–care visits and co-morbidities

Over a period of one year, 35.3% of the asthmatics reported to have sought medical services for respiratory symptoms, 2.2% were hospitalised and 10.6% reported emergency care visits, while the corresponding percentage in the RDF control group was 9.5%, 0.1% and 1.3% respectively.

The studied asthmatic population also outlined for the presence of respiratory comorbidities, characterised by the use of asthma medications and the level of asthma control that the subjects have maintained for the last 12 months (Table 5). Physician diagnosed chronic bronchitis was present in 11.3% of the asthma group, chronic obstructive pulmonary disease was reported by 5.8% and emphysema by 0.7%.

**Table 5:** **Prevalence of comorbidities, use of asthma medications and the level of asthma control (according to GINA guidelines) in the asthmatic population**

Variables	No	Prevalence No (%)
Physician diagnosed comorbidities	744	
COPD		43 (5.8)
Chronic bronchitis		84 (11.3)
Emphysema		5 (0.7)
Level of control (GINA 2006)	669	
Controlled		278 (41.6)
Partly controlled		272 (40.6)
Uncontrolled		119 (17.8)
Exacerbations ≥ 1	744	151 (20.3)
Asthma medications	744	651 (87.5)

### Medications and asthma control

**Figure 6:**
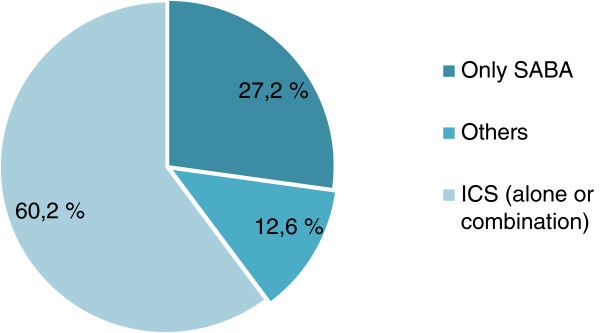
**Proportions of different asthma medications used in the last 12 months.** 87.5% of the asthmatics used some type of asthma medication. SABA – Short acting beta-agonist, ICS – inhaled corticosteroids which includes only ICS, ICS + SABA, ICS and LABA (separately or in a fixed combination), only fixed combination, fixed combination and SABA, Others – only LABA, anticholinergics, antileukotrienes, oral steroids, theophylline and different combinations between them.

From the asthmatics, 87.5% reported using some type of asthma medication (Figure 6). Inhaled corticosteroids (ICS) used alone or in combination with SABA and/or LABA, represented 60.2% of the utilised medications. SABAs as a sole treatment modality were used by 27.2% of the asthmatics. In respect to the level of asthma control, subjects were designated as having controlled, partly controlled or uncontrolled disease according to Global Initiative for Asthma (GINA) 2006 criteria. 41.6% of the asthmatics had controlled disease, still 40.6% were partly controlled and 17.8% of an uncontrolled asthma. We also evaluated how frequent different types of cough were present in the asthmatic subjects who were stratified on the level of control (Table 6). Morning cough was present in the 46% of the partly controlled and 68.1% in the uncontrolled subjects, productive cough – in 38.2% and 68.1% in these groups, respectively and longstanding cough in 33.1% of the partly and 55.5% of the uncontrolled asthmatics.

**Table 6:** **Prevalence of cough in differently controlled asthmatics**

Number of subjects 669	Level of control according to GINA 2006 Guidelines		
Symptoms	Controlled (No 278)	Partly controlled (No 272)	Uncontrolled (No 119)
	Prevalence in the groups%		
Longstanding cough	18.0	33.1	55.5
Sputum production	17.3	38.2	68.1
Sputum production ≥ 3 months	11.8	26.2	49.6
Morning cough	25.9	46.0	68.1

Half of the uncontrolled and one third of partly controlled subjects are troubled by different types of cough.

## Discussion

The West Sweden Asthma Study provides one of the most current descriptions of the state of respiratory symptoms and asthma at a population level in northern Europe [5]. The strength of the study is that it reinforces the concept of supplementing data about subjective symptoms from self-administered questionnaires, with objective data from clinical examinations. This representative cohort of asthmatics had lower lung function than respiratory disease-free subjects, but on average the loss of lung function was small [23]. Nevertheless, around 13% of the asthmatics had persistent airflow limitation and post – bronchodilator FEV1/FVC ratio below 0.70. In some of these subjects this could be due to overlapping COPD, while in others it might be due to variable obstruction without a well-defined cause [24]. Although a greater percentage of asthmatics had increased airway responsiveness, also 30 percent of the respiratory disease-free subjects responded to methacholine. It is well known that airway hyperresponsiveness can be observed also in non-asthmatics, including individuals with allergic rhinitis and atopy [25–27], and in smokers [28, 29], but can also be a predictor for future development of asthma. In the respiratory disease-free subjects, 45% of those who reacted positively to methacholine challenge reported allergic rhinitis and 15% were current smokers which could to some extend account for the increased airway responsiveness. In regard to allergic sensitisation, the greater proportion of the asthmatics showed positive skin-prick reaction to at least one allergen compared to one third of the tested control subjects. Also, asthmatics were far more frequently sensitised to more than four allergens, results which are consistent with previous studies on asthma with different level of severity [17, 30]. In the group of asthmatics, individuals with obesity were significantly more than the individuals in the control group which is in accordance with the bidirectional link which has been speculated between asthma and obesity [31, 32].

A considerable proportion of the subjects from the asthmatic sample were troubled by respiratory symptoms in the year preceding their examination, with the most frequent ones being wheezing with or without shortness of breath, morning and productive cough, shortness of breath and longstanding cough. Furthermore, many asthmatics presented with symptoms that were overlapping, supporting the concept that asthma is a complex and clustered syndrome [33]. We also focused our analyses on different types of cough and their relation with other symptoms and level of asthma control. The asthmatics reported having productive cough always in the presence of longstanding or morning cough and these complaints were broadly superimposed upon each other as well as on wheezing and shortness of breath. By contrast, the control individuals, apart from reporting cough much less frequently, often reported different cough types separately, resulting in morning and longstanding cough without other respiratory symptoms being more frequent in the control group than in the group of the asthmatics. It can only be speculated that some of the latter subjects might fall into the group of cough-variant asthma, but the cross-sectional design of the study precludes us from making such conclusions [34]. Moreover, when we examined the risk factors for partly and uncontrolled disease we found that productive, longstanding and morning cough were highly significant contributors to unsatisfactory controlled disease in the asthmatic group.

Subjects from the asthma group were also classified as having controlled, partly controlled or uncontrolled asthma according to GINA guidelines. Accordingly, around 60% of the asthmatics had inadequately controlled disease and 20% had one or more exacerbations in the last year. The prevalence of longstanding, morning and productive cough increased with the decline of the level of control so that ultimately around 70% of the uncontrolled asthmatics were troubled by morning and productive cough and 55% by longstanding cough. It has been recently proposed that cough frequency can be used as a surrogate marker of asthma control and that “'uncontrolled' asthma patients have significantly higher cough rates than those 'partly controlled' or 'controlled'” [35], a notion that is reinforced by our results. However, further case-control longitudinal studies controlling for co – morbidities are required to support this conjecture.

In our study sample, 87.5% of the asthmatics utilised some type of asthma medication during the last 12 months. The mainstream of asthma treatment is guided by the timely and adequate use of inhaled corticosteroids solely or in combination with bronchodilators. Sixty percent of the asthmatic subjects that we studied were indeed reporting use of some form of ICS, results that correspond to the data from the general population [36]. Asthma management guidelines recommend the use of SABAs to relieve bronchospasm during acute exacerbation and prevent exercise – induced bronchospasm and they should only be used on “as – needed” basis and in the minimal required dose [8]. However, our results show that nearly 30 percent of the asthmatics used solely inhaled short-acting beta - agonist which comprised of around 15% of the uncontrolled and 20% of the partly controlled asthmatics. These results put stress on the necessity of closer monitoring of asthma medication regimens and compliance.

In the present study we opted for more stringent inclusion criteria of the asthmatic individuals with ongoing asthma focusing on those who reported “recurrent wheeze” and not only “any wheeze” in the last 12 months, which can be considered more rigorous than other epidemiological studies evaluating “current asthma” [37–39]. Our approach therefore most likely comprises a slightly more severe group of asthmatics. Additionally, it is also possible that individuals with perhaps more severe and less controlled disease are more prone to attend to the clinical investigation, which could results in an overrepresentation of this group. However, any such effect on prevalence may not be extensive, since our data is in accordance with previous studies from Europe [40, 41]. A cross-sectional study like ours provide important information about present asthma prevalence and how symptoms and other factors overlap, but follow up studies will give more information about time trends and current risk factors of disease progress.

In conclusion, the results from the present study provide the most up-to-date report on asthma symptoms in Northern Europe, and specifically how traditional asthma symptoms and cough overlap. Importantly, approximately 60% of the asthmatics have insufficient control of their disease according to GINA criteria, which may correspond to inadequate utilisation of asthma medications as well as difficult to treat disease. Importantly, cough is relatively more common among asthmatics with signs of more severe disease and should be thoroughly considered when phenotypes of asthma are characterised.

## Abbreviations

FEV1: Forced expiratory volume in 1 second
AR: Allergic rhinitis
SABA: Short-acting beta-2 agonists
LABA: Long-acting beta-2 agonists
ICS: Inhaled corticosteroids
RDF: Respiratory disease – free
GINA: Global Initiative for Asthma.
